# Changes of Blink Reflex in Type 2 Diabetes Mellitus

**DOI:** 10.1155/2021/2473193

**Published:** 2021-03-12

**Authors:** Li Xiao, Kang Zou, Duoyan Zhou, Guilan Ouyang, Shuixiang Liu, Jun Luo

**Affiliations:** ^1^Department of Rehabilitation, The Second Affiliated Hospital of Nanchang University, Nanchang, Jiangxi Province, China; ^2^Department of Neuroelectrophysiology, The First Affiliated Hospital of Gannan Medical University, Ganzhou, Jiangxi Province, China; ^3^Intensive Care Unit, The First Affiliated Hospital of Gannan Medical University, Ganzhou, Jiangxi Province, China

## Abstract

Blink reflex provides an objective assessment of the cranial and central nervous systems. However, the relationships between body mass index, dizziness, and BR have not been explored in patients with type 2 diabetes mellitus (T2DM). Moreover, R2 duration, one of the parameters of the blink reflex, has not been studied to date. In the present study, we aimed to investigate the characteristics and influencing factors of blink reflex in patients with T2DM. We included 45 healthy subjects and 105 hospitalized patients with T2DM. The relationships between these parameters and sex, age, body mass index, duration of T2DM, hemoglobin A1c, distal symmetrical polyneuropathy (DSPN), and dizziness symptoms were analyzed. The results showed that blink reflex latencies (including R1, ipsilateral R2, and contralateral R2 latency) were negatively associated with body mass index but were positively correlated with the duration of T2DM. There were no correlations between blink reflex parameters and sex, age, and hemoglobin A1c. Patients with DSPN had longer blink reflex latencies and shorter R2 durations than those without DSPN. Patients with dizziness had longer latencies (including R1, ipsilateral R2, and contralateral R2 latencies) and shorter R2 durations (including ipsilateral R2 and contralateral R2 durations) than those without dizziness. R2 duration was also a predictive factor for blink reflex abnormality. R2 latency was the most sensitive factor and the optimal predictor of dizziness. These results demonstrate that patients with T2DM with low body mass index, longer duration of T2DM, DSPN, and dizziness-related symptoms had more abnormal blink reflex parameters, indicating more serious injuries to the cranial nerves or the central nervous system.

## 1. Introduction

The complications of diabetes can involve multiple organs and systems including the nervous system. Diabetic peripheral neuropathy is the most common complication of the nervous system, with an incidence of 5%–66% [1]. Diabetic peripheral neuropathy includes autonomic and somatic neuropathy. Diabetic somatic neuropathy includes generalized neuropathy [e.g., diabetic distal symmetrical polyneuropathy (DSPN)] and focal and multiple neuropathy (e.g., cranial neuropathy) [2]. Peripheral nervous system damage is particularly frequent in patients with diabetes, whereas cranial neuropathy is relatively rare [3]. Previous electrophysiological studies mainly focused on limb-associated nerve conduction velocity and F wave, with few studies focusing on cranial nerves and the central nervous system. Therefore, early central nervous system damage in patients with diabetes is more likely to be ignored and misdiagnosed [4].

The blink reflex serves as an objective indicator of the cranial and central nervous systems and their conduction pathways [5]. Previous studies have reported on blink reflex (BR) in diabetic patients and normal healthy subjects and provided a classification of patients with peripheral neuropathy to explore changes in the blink reflex among patients with DSPN [6–8]. However, the relationship between body mass index and the blink reflex has not been determined. In addition, R2 duration, one of the parameters of the blink reflex, has not been studied to date. Finally, the characteristics of blink reflex in patients with diabetes and dizziness have not been explored.

In this study, we systematically analyzed the characteristics and influencing factors of blink reflex in 105 patients with T2DM and discussed the characteristics of blink reflex in those with dizziness.

## 2. Materials and Methods

### 2.1. Study Participants

In this cross-sectional study, we recruited 45 healthy subjects and 105 patients with T2DM admitted to the First Affiliated Hospital of Gannan Medical University between September 2019 and February 2020. All subjects had clear consciousness and could cooperate in the study. The diagnostic criteria and classification of T2DM provided by the World Health Organization in 1999 were adopted to define the diabetic condition [9]. Patients with a history of stroke, cranial nerve lesions, or other diseases related to polyneuropathy or drug-induced neuropathy were excluded. A total of 13 T2DM patients with dizziness were not accompanied by other diseases such as otogenic disease, intracranial tumor, intracranial infection, multiple sclerosis, epilepsy, anemia, and hypertension, which could cause dizziness. Written informed consent was obtained from all included patients.

The study was approved by the Medical Ethics Committee of the First Affiliated Hospital of Gannan Medical University (approval number LLSC-20190801) and was conducted according to the ethical standards of the 1964 Declaration of Helsinki and its later amendments.

### 2.2. Baseline Clinical and Laboratory Measurement

A detailed medical history was obtained from the patients and their families using standardized questions. Demographic data, including sex, age, height, body weight, body mass index, duration of T2DM, and dizziness-related symptoms, were obtained by interview and confirmed by checking patient records. Glycosylated hemoglobin (HbA1c) level was measured after 12 h overnight fasting.

### 2.3. Blink Reflex and Nerve Conduction Study

Blink reflex analysis and nerve conduction studies in the limbs were conducted by an experienced electromyography physician. The patients were tested at the Department of Neuroelectrophysiology, the First Affiliated Hospital of Gannan Medical University. Testing was performed with an electromyography device (MED-9404c; Optoelectronics Corporation, Japan). Room temperature was 25°C–28°C, and body temperature was not less than 32°C. The patient lied in the supine position and relaxed, while breathing calmly with eyes slightly closed. The blink reflex parameter settings were as follows: stimulation frequency of 1 Hz, pulse width of 0.2 ms, bandpass of 2–10 kHz, stimulation intensity of 5 to 10 mA, left and right stimulation (intensity of stimulation was the same), and stimulation interval of 5–10 s. Recording and reference electrodes were attached to the skin at the middle of the orbicularis oculi muscles of both lower eyelids and the temporal side of the outer corner of the eye, respectively. The ground wire was tied to the wrist. Left and right supraorbital incisurae were stimulated 3 to 5 times. Stable and reproducible waveforms were measured, and latencies (including R1, ipsilateral R2, and contralateral R2 latencies) and durations (including ipsilateral R2 and contralateral R2 durations) were recorded.

The nerve conduction study test items included motor and sensory nerve conduction velocities, terminal latency, F wave, and amplitude of the median nerve and ulnar nerve; motor conduction velocity, terminal latency, F wave, and amplitude of the tibial nerve and peroneal nerve; and sensory conduction velocity and amplitude of the sural nerve. The test methods and reference values have been described by Jingxia [10].

### 2.4. Statistical Analysis

The data were analyzed using SPSS 22.0. Normally distributed data are expressed as mean ± standard deviation and nonnormally distributed data as median (quartile). Continuous variables that conformed to a normal distribution were compared using the independent sample *t*-test or analysis of variance. The chi-square test was used to analyze the counting data. Correlation analyses were performed to explore the relationship between blink reflex parameters and variables including age, body mass index, duration of diabetes, and HbA1c level in patients with T2DM. Logistic regression analysis and receiver operating characteristic (ROC) curves were generated for blink reflex parameters in the presence of dizziness. The cutoff value was based on the maximum value of the Youden index. All statistical tests were two tailed, and *P* values of <0.05 were considered to indicate a statistically significant difference.

## 3. Results

### 3.1. General Characteristics of the Study Patients

In this study, 45 healthy subjects and 105 patients with T2DM were recruited. Thirteen patients (12.4%) had dizziness-related symptoms with T2DM. Details about other characteristics including sex, age, body mass index, duration of T2DM, HbA1c level, R1 latency, ipsilateral R2 latency, contralateral R2 latency, ipsilateral R2 duration, and contralateral R2 duration are shown in Table 1. There was no significant difference in sex or age between the healthy and T2DM groups (*P* > 0.05 and *P* > 0.05, respectively). However, R1, ipsilateral R2, and contralateral R2 latencies were different between the two groups (all *P* ≤ 0.001). Differences in ipsilateral R2 and contralateral R2 duration between the two groups were not significant (*P* > 0.05). In 105 patients with T2DM, there were no significant sex-specific differences in terms of any of these characteristics (all *P* > 0.05; Table 2).

### 3.2. Correlation Analysis

Correlations were analyzed between age, body mass index, duration of T2DM, and HbA1c levels and blink reflex parameters (Table 3). Notably, blink reflex latencies (including R1 latency, ipsilateral R2 latency, and contralateral R2 latency) were negatively associated with body mass index (all *P* < 0.05) but were positively correlated with the duration of T2DM (all *P* < 0.001). In addition, regarding body mass index and duration of T2DM, the absolute correlation coefficient values of ipsilateral R2 latency (*r* = −0.185 and 0.28) and contralateral R2 latency (*r* = −0.182 and 0.256) were higher than those of R1 latency (*r* = −0.158 and 0.184). Ipsilateral R2 duration was negatively associated with the duration of T2DM (*P* < 0.05). Age and HbA1c levels were not correlated with any blink reflex parameter (*P* > 0.05).

### 3.3. Differences in Blink Reflex Parameters between the Normal, T2DM without DSPN, and T2DM with DSPN Groups

There were significant differences in blink reflex latencies (including R1 latency, ipsilateral R2 latency, and contralateral R2 latency) between normal, T2DM without DSPN, and T2DM with DSPN groups (all *P* < 0.01). Moreover, there were significant differences in blink reflex durations (including ipsilateral R2 and contralateral R2 duration) between the T2DM with DSPN group and the other two groups (both *P* ≤ 0.001), but the differences in blink reflex duration between the normal group and the T2DM without DSPN group were not significant (both *P* > 0.05; Table 4).

### 3.4. Evaluation of Blink Reflex Parameters according to the Presence of Dizziness

The findings of blink reflex parameters were significantly different between patients with dizziness and those without dizziness (*P* = 0.002 or *P* ≤ 0.001; Table 5).

The differences between two-side R2 latencies (*P* = 0.794) and between two-side R2 durations (*P* = 0.544) were not significant. In addition, determination of collinearity by regression analysis between two-side R2 latencies and between two-side R2 durations showed that the degree of tolerance was very small (0.123, 0.213) and the variance inflation factor value was large (8.121, 4.687). Thus, substantial collinearity was determined between them. Therefore, in the following logistic regression analysis and ROC analysis, we eliminated contralateral R2 latency and contralateral R2 duration and only analyzed the relationship of dizziness with R1 latency, ipsilateral R2 latency, and ipsilateral R2 duration.

Binary logistic regressions were performed for the correlation between dizziness-related symptoms and R1 latency, ipsilateral R2 latency, and ipsilateral R2 duration.  Table 6 shows the correlation between dizziness-related symptoms and ipsilateral R2 latency and ipsilateral R2 duration (*P* = 0.011 and *P* ≤ 0.001, respectively). Ipsilateral R2 latency had a high odds ratio (OR, 1.440; 95% CI, 1.226–1.692), whereas ipsilateral R2 duration had a low OR (OR, 0.899; 95% CI, 0.828–0.976). No significant correlation was observed between dizziness-related symptoms and R1 latency (*P* = 0.982).

**Figure 1:**
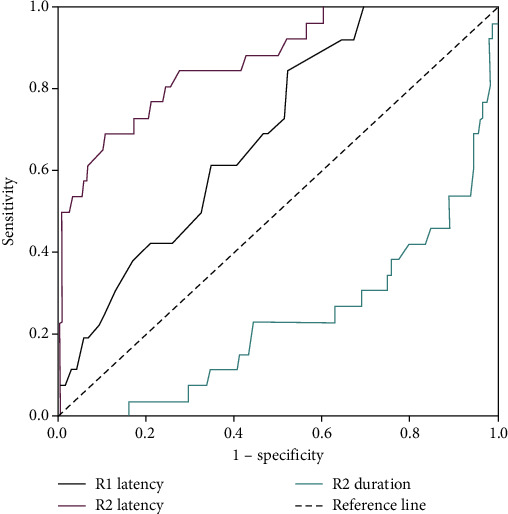
Graph ROC curves showing AUCs of different BR parameters for the diagnosis of dizziness. Abbreviations: AUC: area under the curve; ROC: receiver operating characteristic.

Area under the curve (AUC), sensitivity, and specificity were assessed for the diagnostic value of dizziness for each blink reflex parameter (Table 7). All parameters revealed predictive capabilities (AUC > 0.5; Figure 1). Ipsilateral R2 latency yielded the highest AUC among all examined parameters at 0.867 (95% CI, 0.792 to 0.943), followed by ipsilateral R2 duration (0.768; 95% CI, 0.666 to 0.870) and IR1 latency (0.692; 95% CI, 0.597–0.786). The cutoff value was 37.65 for ipsilateral R2 latency (sensitivity, 69.2%; specificity, 89.1%), 38.05 for ipsilateral R2 duration (sensitivity, 75.0%; specificity, 69.2%), and 10.45 for R1 latency (sensitivity, 84.6%; specificity, 47.8%).

## 4. Discussion

**Figure 2:**
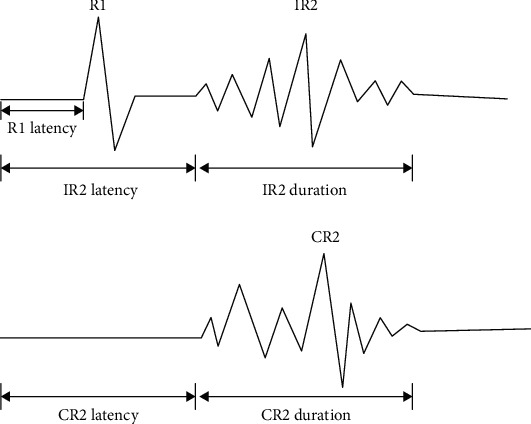
Schematic of blink reflex. Abbreviations: IR2: ipsilateral R2; CR2: contralateral R2.

The waveform of the blink reflex consists of two main components, R1 and R2 (including IR2 and CR2), as shown in Figure 2. R1 has an early phase. In most cases, its waveform is sharp and single, with good repeatability. Its pathway is that the nerve impulse is transmitted to the trigeminal sensory nucleus of the pons through the trigeminal nerve branch, connected with the ipsilateral nerve nucleus through adjacent interneurons, and then transmitted through the facial nerve. The whole process involves only 1 to 3 interneurons [11]. IR2 and CR2 are relatively late, and they are multiphase waves with various waveforms. The pathway is that nerve impulses first enter the trigeminal cord of the ipsilateral pons and lateral medulla oblongata via trigeminal nerve branches and then descend to the tail of the medulla oblongata (i.e., at the level of the lower olivary nucleus and the tail of the sublingual nerve nucleus) and form synapses with the trigeminal spinal tract nucleus. The fibers from the trigeminal spinal tract nucleus go up through the multisynaptic pathway in the lateral network structure of the medulla oblongata, divide branches in the adjacent pons and the medulla oblongata, reach the bilateral nerve nucleus, and then pass out through the facial nerve [12, 13]. Therefore, the pathway of the blink reflex involves the peripheral nerve (trigeminal nerve, facial nerve) and central nerve (pons, medulla oblongata). We can evaluate the damage degree of this pathway (including peripheral and central nerves) by analyzing the parameters of the bilateral blink reflex.

In the current study, we systematically analyzed the characteristics and assessed the influencing factors of the blink reflex (including body mass index, duration of diabetes, and DSPN) in patients with T2DM. We found that R2 duration was also a predictive factor for blink reflex abnormalities. In addition, we confirmed that there were significant differences in blink reflex parameters between patients with dizziness and those without dizziness, which showed that patients with dizziness had longer latencies (including R1 ipsilateral R2 and contralateral R2 latency) and shorter R2 durations (including ipsilateral R2 and contralateral R2 duration). Finally, our findings showed that R2 latency was the most sensitive factor and yielded the optimal predictive value for dizziness compared with the other blink reflex parameters.

First, we compared the sex and age of 45 healthy individuals and 105 T2DM patients and found no statistical difference. Subsequently, we compared the parameters of blink reflex between the two groups. We found that the latencies of blink reflex (including R1, ipsilateral R2, and contralateral R2 latency) in patients with T2DM were longer than those in healthy individuals. These findings were consistent with those reported by Kazem and Behzad [14] and Y. Lian-hong [15], but were inconsistent with those reported by Nazliel et al. [16]. However, we found no difference in ipsilateral R2 or contralateral R2 duration between the two groups.

Our study showed that body mass index was negatively associated with blink reflex latencies (including R1 latency, ipsilateral R2 latency, and contralateral R2 latency). In other words, the lower the body mass index, the more abnormal the blink reflex. This phenomenon is consistent with the view that low body mass index exists in patients with poorly controlled T2DM [17, 18].

We found that the duration of T2DM was positively correlated with blink reflex latency OR, and the correlation with R2 latency was greater than that with R1 latency. These findings were consistent with those reported by Elkholy et al. [19], but were inconsistent with those reported by Kazem and Behzad [14] and Kiziltan et al. [20], who found that the correlation was higher for R1 latency. These differences may be due to differences in factors such as sample size, patient selection, and detection methods. However, no significant correlations were observed between blink reflex parameters and sex, age, or HbA1C levels. The results of our study were consistent with those of prior reports [16, 21].

In previous studies, patients with T2DM were divided into two groups according to the presence of diabetic neuropathy. R1 latency, ipsilateral R2 latency, and contralateral R2 latency were prolonged in all diabetic patients with or without polyneuropathy compared with in controls [22]. Patients with DSPN had longer R1 latency, ipsilateral R2 latency, and contralateral R2 latency than those without DSPN [6]. The results of our study were consistent with those of previous investigations. Moreover, we also found that R2 durations (including ipsilateral R2 and contralateral R2 durations) of patients with DSPN were shorter than those of normal subjects and diabetic patients without DSPN. There is a multisynaptic connection between the reflex arc of R2 and the intermediate neurons of the reticular structure, which is susceptible to many factors such as lesions of the thalamus and brain as well as mental state [23]. In addition, the shortening of ipsilateral R2 and contralateral R2 durations indicates that the number of interneurons associated with multisynaptic reflex activity and excitability decreased [24]. Therefore, not only the latency of the blink reflex but also R2 duration can reflect the degree of lesions of the central nervous system such as the brainstem, thalamus, and brain in patients with T2DM.

We found that blink reflex parameters in patients with dizziness were significantly different from those in patients without dizziness, with the former having longer latencies (including R1, ipsilateral R2, and contralateral R2 latencies) and shorter durations (including ipsilateral R2 and contralateral R2 durations). To further investigate the relationship between dizziness and the blink reflex, we performed logistic regression analysis to show that R2 latency had the highest OR, which indicated that R2 latency had the highest predictive value for dizziness among all blink reflex parameters. Using ROC analysis, we concluded that R2 latency is the most sensitive indicator of dizziness-related symptoms.

This study has some limitations. First, the study was performed at a single center. Second, it was only a cross-sectional observational study and did not prove causality. Lastly, patient selection bias might exist.

## 5. Conclusions

In conclusion, blink reflex parameters (including R1 latency, ipsilateral R2 latency, contralateral R2 latency, ipsilateral R2 duration, and contralateral R2 duration) can help to evaluate injuries of the cranial nerves and the central nervous system in patients with T2DM.

## Figures and Tables

**Table 1:** Clinical characteristics of the study participants.

Characteristics	Normal (*n* = 45)	T2DM (*n* = 105)	*P* value
Sex (*n*, %)^†^			0.081
Male	17 (37.8)	56 (53.3)	
Female	28 (62.2)	49 (46.7)	
Age (year)^‡^	53.13 ± 9.04	55.30 ± 12.23	0.091
R1 latency (ms)^‡^	9.76 ± 0.60	10.81 ± 0.91	*P* ≤ 0.001^∗∗^
IR2 latency (ms)^‡^	29.82 ± 3.57	33.54 ± 4.88	*P* ≤ 0.001^∗∗^
CR2 latency (ms)^‡^	29.73 ± 3.83	33.23 ± 5.08	*P* ≤ 0.001^∗∗^
IR2 duration (ms)^‡^	44.12 ± 7.67	42.84 ± 9.36	0.253
CR2 duration (ms)^‡^	44.73 ± 7.44	43.06 ± 8.96	0.120
BMI (kg/m^2^)	—	24.04 ± 3.06	—
DM duration (year)	—	5 (10)	—
HbA1C (%)	—	9.96 (3.60)	—
Dizziness (*n*, %)	—	13 (12.4)	—

^†^Analysis of Pearson chi-square test. ^‡^Analysis of independent sample *t*-test. ^∗∗^*P* ≤ 0.001. Abbreviations: *n*: number of cases; mean ± SD: mean ± standard deviations; 95% CI: 95% confidence interval; BMI: body mass index; HbA1c: hemoglobin A1c; IR2: ipsilateral R2; CR2: contralateral R2.

**Table 2:** Differences in blink reflex between different sexes in patients with T2DM.

	Male (*n* = 56)	Female (*n* = 49)	*t*-value	*P* value
R1 latency (ms)	10.92 ± 0.96	10.68 ± 0.82	1.96	0.51
IR2 latency (ms)	33.81 ± 4.98	33.22 ± 4.74	0.886	0.376
CR2 latency (ms)	33.70 ± 5.22	32.71 ± 4.86	1.408	0.161
IR2 duration (ms)	42.19 ± 7.90	43.79 ± 10.93	−1.189	0.236
CR2 duration (ms)	41.99 ± 7.56	44.47 ± 10.16	−1.963	0.051

Data are presented as mean ± standard deviation. Abbreviations: *n*: number of cases; IR2: ipsilateral R2; CR2: contralateral R2.

**Table 3:** Correlation analysis of blink reflex with age, BMI, duration of T2DM, and HbA1C.

	R1 latency		IR2 latency		CR2 latency		IR2 duration		CR2 duration	
	*r*	*P* value	*r*	*P* value	*r*	*P* value	*r*	*P* value	*r*	*P* value
Age^†^	0.144	0.141	0.066	0.505	0.058	0.559	−0.065	0.509	−0.008	0.935
BMI^†^	−0.158	0.022^∗^	−0.185	0.007^∗^	−0.182	0.008^∗^	0.113	0.102	0.119	0.085
T2DM duration^‡^	0.184	0.008^∗^	0.28	*P* ≤ 0.001^∗∗^	0.256	*P* ≤ 0.001^∗∗^	−0.156	0.025^∗^	−0.129	0.064
HbA1C^‡^	−0.113	0.118	0.057	0.435	0.034	0.638	0.023	0.755	0.017	0.817

^†^Person correlation analysis for normally distributed data. ^‡^Spearman correlation analysis for nonnormally distributed data. *r*: correlation coefficient. ^∗^*P* < 0.05, ^∗∗^*P* ≤ 0.001, Abbreviations: IR2: ipsilateral R2; CR2: contralateral R2; BMI: body mass index; HbA1c: hemoglobin A1c.

**Table 4:** Differences in blink reflex among the normal, T2DM without DSPN, and T2DM with DSPN groups.

	Healthy (*n* = 45)	T2DM without DSPN (*n* = 60)	T2DM with DSPN (*n* = 45)	*P* value
R1 latency (ms)^†^	9.76 ± 0.60	10.45 ± 0.73	11.29 ± 0.89	*P* ≤ 0.001
IR2 latency (ms)^‡†^	29.82 ± 3.57	31.70 ± 4.30	35.98 ± 4.51	0.001
CR2 latency (ms)^†^	29.73 ± 3.83	31.45 ± 4.55	35.63 ± 4.75	0.005
IR2 duration (ms)^‡^	44.12 ± 7.67	45.72 ± 9.75	39.22 ± 7.61	*P* ≤ 0.001
CR2 duration (ms)^‡^	44.74 ± 7.44	45.97 ± 9.19	39.38 ± 7.29	*P* ≤ 0.001

^†^ *P* < 0.01 for the pairwise comparison of the healthy, T2DM without DSPN, and T2DM with DSPN groups. ^‡^*P* ≤ 0.001 for the comparison between the T2DM with DSPN group with the other two groups; *P* > 0.05 for the comparison between the normal group and the T2DM without DSPN groups. Abbreviations: IR2: ipsilateral R2; CR2: contralateral R2; DSPN: diabetic distal symmetric polyneuritis.

**Table 5:** Differences in blink reflex between nondizziness and dizziness groups.

	Nondizziness (*n* = 92)	Dizziness (*n* = 13)	*t*-value	*P* value
R1 latency (ms)	10.74 ± 0.90	11.33 ± 0.86	3.182	0.002^∗^
IR2 latency (ms)	32.70 ± 4.35	39.47 ± 4.22	7.454	*P* ≤ 0.001^∗∗^
CR2 latency (ms)	32.40 ± 4.62	39.17 ± 4.05	7.09	*P* ≤ 0.001^∗∗^
IR2 duration (ms)	43.95 ± 9.28	35.72 ± 7.30	−4.335	*P* ≤ 0.001^∗∗^
CR2 duration (ms)	44.00 ± 8.95	37.12 ± 7.18	−3.751	*P* ≤ 0.001^∗∗^

^∗^ *P* < 0.01, ^∗∗^*P* ≤ 0.001. Abbreviations: *n*: number of cases; IR2: ipsilateral R2; CR2: contralateral R2.

**Table 6:** Odds ratio for increasing risk of dizziness in binary logistic regression.

	OR	95% CI	*P* value
R1 latency	0.993	0.549–1.797	0.982
R2 latency	1.44	1.226–1.692	*P* ≤ 0.001^∗∗^
R2 duration	0.899	0.828–0.976	0.011^∗^

^∗^ *P* < 0.05, ^∗∗^*P* ≤ 0.001. Abbreviations: OR: odds ratio; 95% CI: 95% confidence interval.

**Table 7:** Evaluation of blink reflex parameters in the diagnosis of dizziness.

	AUC (95% CI)	Cutoff	Sensitivity (%)	Specificity (%)	*P* value
R1 latency	0.692 (0.597–0.786)	10.45	84.6	47.8	0.002^∗^
R2 latency	0.867 (0.792–0.943)	37.65	69.2	89.1	*P* ≤ 0.001^∗∗^
R2 duration	0.768 (0.666–0.870)	38.05	75	69.2	*P* ≤ 0.001^∗∗^

^∗^ *P* < 0.01, ^∗∗^*P* ≤ 0.001. Abbreviations: AUC: area under the curve; 95% CI: 95% confidence interval.

## Data Availability

The [DATA.pdf] data used to support the findings of this study is included within the supplementary information file.

## Supplementary Materials

Supplementary Materials The materials in the excel sheet are raw data which are used to derive the data in the tables and figures in the manuscript.
