# Supplementary material for: Changes of Blink Reflex in Type 2 Diabetes Mellitus
Source: J Diabetes Res. 2021 Mar 12;2021:2473193. doi: 10.1155/2021/2473193 (PMC7984919; doi:10.1155/2021/2473193)
Supplement: Supplementary Materials — The materials in the excel sheet are raw data which are used to derive the data in the tables and figures in the manuscript. [file 2473193.f1.zip › Supplementary Description[1].docx]

**Supplementary Description:**

The materials in excel sheet is an raw data which is used to derive the data in tables and figures in the manuscript
